# Evidence on scaling in health and social care: protocol for a living umbrella review

**DOI:** 10.1186/s13643-021-01813-3

**Published:** 2021-09-30

**Authors:** France Légaré, Karine V. Plourde, Ali Ben Charif, Amédé Gogovor, Francesca Katherine Brundisini, Robert K. D. McLean, Andrew Milat, Nathalie Rheault, Luke Wolfenden, Hervé Tchala Vignon Zomahoun

**Affiliations:** 1 VITAM – Centre de recherche en santé durable, Université Laval, Pavillon Landry-Poulin – 2525, Chemin de la Canardière, Quebec, QC Canada; 2 Tier 1 Canada Research Chair in Shared Decision Making and Knowledge Translation, Université Laval, Quebec, QC Canada; 3 Health and Social Services Systems, Knowledge Translation and Implementation component of the Quebec SPOR-SUPPORT Unit, Université Laval, Quebec, QC Canada; 4 Department of Family Medicine and Emergency Medicine, Université Laval, Quebec, QC Canada; 5 International Development Research Centre, Ottawa, ON Canada; 6 Faculty of Medicine and Health Sciences, Stellenbosch University, Tygerberg, South Africa; 7 School of Public Health, University of Sydney, Sydney, NSW Australia; 8 Centre for Epidemiology and Evidence, NSW Ministry of Health, Sydney, NSW Australia; 9 School of Medicine and Public Health, University of Newcastle, Callaghan, NSW Australia; 10 Hunter Medical Research Institute, New Lambton Heights, NSW Australia; 11 Hunter New England Population Health, Wallsend, NSW Australia

**Keywords:** Scaling, Spread, Scalability, Implementation science, Scaling science, Knowledge translation, Health and social care, Living review, Umbrella review

## Abstract

**Background:**

There is a growing interest in scaling effective health innovations to promote equitable access to high-quality health services worldwide. However, multiple challenges persist in scaling innovations. In this study, we aim to summarize the scaling evidence in the health and social care literature and identify current knowledge gaps.

**Methods:**

We will conduct a living umbrella review according to the Joanna Briggs Institute Reviewers’ Manual. We will consider all knowledge syntheses addressing scaling in health or social care (e.g., any setting, any clinical area) and conducted in a systematic way. We will search the following electronic databases: MEDLINE (Ovid), Embase, PsychINFO (Ovid), CINAHL (EBSCO), Web of Science, The Cochrane Library, Sociological Abstract (Proquest), Academic Search Premier (EBSCO), and Proquest Dissertations & Theses Global, from inception. Furthermore, we will conduct searches of the grey literature. No restriction regarding date or language will be applied. Each phase of the review will be processed by two independent reviewers. We will develop a data extraction form on Covidence. We will assess the methodological quality of the included reviews using AMSTAR2 and the risk of bias using ROBIS. Results will be presented in tabular form and accompanied by a narrative synthesis covering the traditional themes of scaling science that emerge from the analysis, such as coverage, range, and sustainability, as well as themes less covered in the literature, including reporting guidance, models, tools, barriers, and/or facilitators to scaling innovations, evidence regarding application in high-income or low-income countries, and end-user engagement. We will disseminate the findings via publications and through relevant networks.

**Discussion:**

The findings of the umbrella review will facilitate access to scaling evidence in the literature and help strengthen the science of scaling for researchers, policy makers, and program managers. Finally, this work will highlight important knowledge gaps and help prioritize future research questions.

**Systematic review registration:**

This protocol was registered with the International Prospective Register of Systematic Reviews (PROSPERO) on November 11, 2020 (registration number: CRD42020183774).

**Supplementary Information:**

The online version contains supplementary material available at 10.1186/s13643-021-01813-3.

## Background

There is great interest in implementing health care innovations at a larger scale to achieve better health of populations and to reduce per capita cost of health care. Spread (replicating an innovation) and scale (building infrastructure to support full scale implementation) [1] are both used in the fields of knowledge translation (KT) and implementation science to refer to increasing the reach and adoption of innovations. The concepts of spread and scale broadly correspond with what others describe respectively as vertical and horizontal approaches. Vertical scaling up consists of using policy, regulation, or financial tools to expand an innovation simultaneously across a whole system (e.g., introduction of mandatory seatbelt legislation), while horizontal scaling up is the phased expansion, often starting with a pilot project, of an innovation (e.g., a lifestyle-based diabetes program) into more care settings [2–4]. Canada’s International Development Research Centre (IDRC) has suggested the notion of “scaling science” as a complementary domain to implementation science or KT science that is specifically concerned with the optimization of the magnitude, variety, equity, and sustainability of health and social impacts [5–7]. In this review, we define “scaling” as inclusive of all variants of spread, scale or scaling up, scaling out or scaling deep, horizontal, and vertical. By adopting a broad view, we aim to cast a wide net and thereby increase the learning potential of the study.

A variety of scaling models have been developed in the health and social care setting in the last 10 years. However, there is a persistent failure to scale innovations across any health care system, whether in high-, middle-, or low-income countries [5, 8]. This failure may be partly due to a lack of scientific knowledge about scaling [5, 9, 10]. An intervention that is proven effective at one scale may have quantitatively or qualitatively new or different impacts at another scale. Research to help predict intervention impacts at scale and to guide the development and execution of scaling strategies is required to improve the success of scaling efforts [5]. Results could support policy makers, program managers, and implementers to identify the right evidence to support their decision-making and plans for scaling.

Many of the existing systematic reviews on scaling in health and social care focus on a specific area of care, and so evidence is fragmented [10–15]. An umbrella review synthesizes the findings of literature reviews already available. Scaling science is moving quickly as new evidence emerges, and thus a living review is also appropriate as it updates the evidence on a continuous basis. We therefore aim to undertake a living umbrella review on scaling science in health and social care. Our proposal satisfies the three guiding criteria for systematic living reviews [16, 17]. Our study will reconcile all sources of evidence, generalize findings from all types of literature review following a systematic approach and provide a single document that summarizes findings. Additionally, it will facilitate access to the literature and help policy makers and program managers make informed decisions about scaling in health and social care. Finally, it will signal important knowledge gaps and help prioritize future research.

## Methods

We adopted the Joanna Briggs Institute (JBI) methodology for umbrella reviews [18], and we used the Preferred Reporting Items for Systematic Reviews and Meta-Analyses-Protocol (PRISMA-P) guidelines to structure this protocol (Additional file 1) [19]. The study protocol was registered with the International Prospective Register of Systematic Reviews (PROSPERO, CRD42020183774).

### Research question

What is the evidence about scaling in health and social care and what are the knowledge gaps in the literature?

### Eligibility criteria

We will address all types of evidence matching the “PICO” criteria (Participants, Intervention, Comparator, Outcome) and “PICo” (Population, phenomena of Interest and Context) to capture the evidence from quantitative and qualitative reviews.

#### Participants or population

All reviews that include primary studies focusing on individuals (e.g., patients or caregivers, health care providers), or health systems, services, or organizations that have been exposed to the scaling of a health or social care innovation.

There will be no restrictions based on socio-demographic factors (e.g., age, ethnicity, socio-economic status) or general health conditions (e.g., comorbidities).

#### Interventions/phenomena of interest

No restrictions. We will consider all types of scaling, vertical and horizontal. This umbrella review will not only explore the scaling of health and social care innovations but also any aspect of or any topic relating to such scaling, including (but not limited to) concepts, models, analytical models, tools, cost or impact assessments, and user engagement.

#### Comparator

No restrictions.

#### Outcomes

We will consider all outcomes reported in the included reviews (i.e., no restrictions), including health outcomes. We will seek outcomes such as (but not limited to) patients/caregivers and/or health care providers and/or policy makers’ perceptions and experiences of barriers, facilitators, acceptability and feasibility of scaling innovations, impact (e.g., adaptability, efficacy, effectiveness), coverage (e.g., proportion of the target population that is reached by the scaling, adoption, fidelity, penetration, or maintenance of the innovation), health outcomes (e.g., impact on morbidity, mortality), patient reported outcomes (e.g., quality of life, satisfaction), and health care resources (e.g., cost-effectiveness of the scaled innovation, cost of staff resources).

#### Types of study

We will consider all types of review (quantitative, qualitative, and mixed-methods) that address, synthesize, and summarize evidence in the field of scaling. We define a review as a knowledge synthesis of evidence that includes a clear research question, describes the methods used (which are reproducible) to identify and select the primary research studies, and synthesizes data from its included studies [20, 21]. Reviews can include studies with any research design. We will include all types of review that have been rigorously conducted according to their chosen methodological approach. We will exclude reviews that do not describe their search strategy and inclusion criteria explicitly at the stage of full-text screening. We will also exclude primary research studies, conference abstracts, comments, opinions, letters, and editorials.

#### Context/setting

We will include reviews regarding any type of health and social care setting (e.g., home care, community, hospital, primary care, specialized care) in any geographical setting (e.g., rural or urban regions, low-, middle- or high-income countries).

We define “health and social care services” as follows:

Health care consists of services provided in institutional or community settings, any form of access to a health-related service (such as dental, podiatry, or optical services), and access to health care practitioners (such as nurses, physiotherapists, or general practitioners) [22].

Social care consists of interventions that support frail or vulnerable individuals by meeting needs or enabling them to meet needs that arise as a result of physical, mental, or emotional impairment [23].

#### Search strategy

Our information specialist (NR) will develop an Ovid-MEDLINE and Web of Science strategy with input from the project team and a second information specialist. An iterative revision process will be conducted by the members of the research team. Research keywords will include the following: “scaling,” “reviews,” and “health and social care”. We will use the search strategy for “scaling up” previously developed by members of our team. The Canadian Agency for Drugs and Technologies in Health (CADTH) search filter for Systematic Reviews/Meta-Analysis/Health Technology Assessment was adapted for this project [24]. A second information specialist will review the search strategy using the Peer Review of Electronic Search Strategies (PRESS) tool [25]. Comments will be integrated in a final version of the search strategy. The final version will be approved by the team members. Once approved, this search strategy will be translated into the other databases mentioned below. The exact search terms will be recorded as the search strategy is refined. This protocol only includes the search strategy conducted in one database (Additional file 2). A further systematic literature search will be performed to identify published studies in the following electronic bibliographic databases: MEDLINE (Ovid), Embase, PsychINFO (Ovid), CINAHL (EBSCO), Web of Science, The Cochrane Library, Sociological Abstract (Proquest), Academic Search Premier (EBSCO), and Proquest Dissertations & Theses Global. No language restriction will be applied. We will search from inception onwards.

Additionally, to identify the grey literature, we will search the websites of relevant organizations such as the World Health Organization (WHO), Global Reporting Initiative, the UK’s National Institute for Care and Excellence (NICE), Australia NSW Government, the Institut National d’Excellence en Santé et Services Sociaux (INESSS) in Quebec, Canada, the Canadian Foundation for Healthcare Improvement (CFHI), Canada’s IDRC, the CADTH’s Grey Matters checklist, and clinical trial registries. We will contact experts in the field by email for additional data. In addition to searching databases, the reference list of each included review will be reviewed.

#### Selection process

The search results will be imported and stored in an EndNote X9 library for reference management and duplicate removal [26, 27]. The resulting records will be exported to the Internet-based system Covidence for the selection process [28]. The team will develop a selection grid. It will be adjusted, if necessary, before the screening of titles and abstracts of all articles. All selection criteria will be discussed by reviewers to ensure common understanding. A pilot screening (titles and abstracts) of 2.5% random sample reviews will be completed. Discrepancies will be resolved by consensus or by a third reviewer if necessary.

First, two independent reviewers will screen titles and abstracts of identified reviews against the eligibility criteria. Articles with abstracts that do not appear to meet the criteria for exclusion, or are ambiguous, or have missing abstracts, will be retained and reviewed in full. Second, full-text examination of the remaining reviews will be assessed for eligibility. The reviewers’ full understanding of the selection criteria will be validated again before beginning this stage. In case of an “unclear” response regarding the eligibility of studies, authors will resolve disagreements through discussion and, if necessary, consult a third senior author. Any reasons for exclusion will be recorded in Excel.

Articles that are not available electronically will be ordered via interlibrary loan. We will contact the corresponding author if an email is available.

#### Data extraction

Once the reviews have been selected for inclusion, two reviewers will independently conduct the data extraction. We will develop a data extraction form based on the JBI form for review of systematic reviews. A standardized, pre-piloted form will be used to extract data from the included reviews for assessment of study quality and evidence synthesis. A pilot data extraction will be conducted on 5% of selected reviews until conclusive results are agreed on between reviewers. Any disagreement between the reviewers will be resolved through discussion; if consensus is not reached, they will consult a third senior reviewer. We will not extract data from the primary studies included in the reviews. We will summarize the review findings but we will not re-synthesize the results of primary studies. Extracted information will include:

- Review characteristics: author, year of publication, country, type of review; aim of the study; language; PICO or PICo; number and characteristics of participants (e.g. sexas a biological variable, age); setting and context; number and type of databases sourced and searched; date range of database searching; citation index; number and study design of primary studies included in each review; instrument used to appraise the primary studies and the rating of their quality; method of synthesis/analysis used to synthesize the evidence; synthesis/summary of results; heterogeneity if applicable; mention by the authors of research needs or gaps in the review.
- Focus of the reviews: infrastructure (e.g., policies, guidelines, human and material resources); concepts and models (e.g., definition, conceptual model, framework); measurement (e.g., tools, scalability, cost); analytical methods (e.g., mathematical approach); descriptive (e.g., perceptions, barriers, facilitators, acceptability); patient engagement (e.g., authorship, study’s reporting on patient advocates) and scaled interventions (e.g., strategies, training, process, coverage, feasibility, effectiveness).

### Assessing quality of reviews

Two independent reviewers will use AMSTAR2 [29] to assess the methodological quality of included reviews and ROBIS [30] for the risk of bias. Based on the nature of the included reviews, we will explore the relevance of using the GRADE assessment system [31, 32]. Disagreements between the reviewers over the quality or the risk of bias in particular studies will be resolved by discussion, with involvement of a third author where necessary. AMSTAR2 is a validated, widely used 16-item instrument for assessing reviews of reviews. ROBIS contains 24 questions divided into three phases. Phase 1 assesses relevance (optional) and verifies if the research question matches the umbrella review’s PICO; phase 2 identifies concerns with the review process; and phase 3 judges risk of bias in the review. Phase 2 and 3 questions are answered with the following options: yes, probably yes, probably no, no, no information. The concerns regarding phase 2 domains and phase 3 are classified as high, low, or unclear.

### Data synthesis

We will use the Preferred Reporting Items for Systematic Reviews and Meta-Analyses (PRISMA) flow diagram to describe the process of study selection [33]. We will use tables to describe characteristics of included studies (e.g., first author’s name, year of publication, study aim) and analysis details (e.g., appraisal instrument used, methods of analysis, results summary, authors comments, heterogeneity of the results), and we will synthesize the data narratively using categories of scaling evidence that have emerged from the included reviews (e.g., descriptive reviews of scaling, reviews of barriers and facilitators, reviews of determinants, reviews of concepts, reviews of frameworks, reviews of economic evaluation, reviews of scaling interventions, reviews of scaling measures, reviews of effects of scaling interventions, reviews of scalability).

A meta-analysis will not be performed, given that pooling the results of qualitative and quantitative reviews can introduce significant overlap and bias. We will clearly indicate the overlap between primary studies within the reviews in the tables, and we will develop a citation matrix. Because our purpose is to identify evidence about scaling in health and social care in the literature, we will present the whole body of knowledge and include the results of all the reviews, regardless of the overlap across primary studies [34].

### Living update

Based on the series Living Systematic Reviews [17, 35–38], the search for new reviews will be repeated at regular intervals by the coordinator team, i.e., tri-monthly searches of bibliographic databases and every six months for the grey literature. The studies identified during these updated searches will be analyzed using the same eligibility criteria as used in the initial search. The new information will be integrated at least once a year, when it will be combined with all the “stand by” information. If the information has an effect on the evidence, it will be included in the review and an update will be published.

## Discussion

This review will help to build the science of scaling in health and social care. We are not aware of any other umbrella or systematic review addressing this issue. As an international group of researchers, policy-makers, and funders, we are aware of the increasing importance of the science of scaling and its potential for optimizing the benefits of health research for individuals, institutions, and systems. Anticipated challenges for this review include the complexity of summarizing the research syntheses from such a diversity of review types and topics and to determine the point when newly identified elements of scaling science can be described as common themes. Important protocol amendments will be documented and noted in the discussion. The dissemination plan is to present the results through publications in peer-review journals, relevant networks and social media, and presentations at national and international conferences.

## Supplementary Information

**Additional file 1.** PRISMA-P (Preferred Reporting Items for Systematic review and Meta-Analysis Protocols) 2015 checklist: recommended items to address in a systematic review protocol*.

**Additional file 2.** Search strategy.

## Data Availability

The datasets used and/or analyzed during the current study are available from the corresponding author on reasonable request.

## Abbreviations

JBI: Joanna Briggs Institute
AMSTAR2: A Measurement Tool to Assess Systematic Reviews 2
ROBIS: Risk of Bias in Systematic Reviews
PROSPERO: International Prospective Register of Systematic Reviews
KT: Knowledge Translation
IDRC: International Development Research Centre
GRADE: Grading of Recommendations, Assessment, Development and Evaluation
PRISMA: Preferred Reporting Items for Systematic Reviews and Meta-Analyses
PRISMA-P: Preferred Reporting Items for Systematic Reviews and Meta-Analyses-Protocol
PICO: Participants, Intervention, Comparator, Outcome
PICo: Population, phenomena of Interest and Context
PRESS: Peer Review of Electronic Search Strategies
WHO: World Health Organization
NICE: National Institute for Care and Excellence (UK)
INESSS: Institut National d’Excellence en Santé et Services Sociaux (Canada)
CFHI: Canadian Foundation for Healthcare Improvement
NSW: New South Wales
CADTH: Canadian Agency for Drugs and Technologies in Health
MLS: Masters in Library Science
CIHR: Canadian Institutes of Health Research
FRQ-S: Fonds de recherche du Québec-Santé
