# Supplementary material for: Evidence on scaling in health and social care: protocol for a living umbrella review
Source: Syst Rev. 2021 Sep 30;10:261. doi: 10.1186/s13643-021-01813-3 (PMC8485425; doi:10.1186/s13643-021-01813-3)
Supplement: Supplementary file 2 — Additional file 2. Search strategy. [file 13643_2021_1813_MOESM2_ESM.docx]

### Medline (Ovid) (2020-08-06)

| **Concepts** | **Search strategy keywords** | **Search** | **# Results** |
| --- | --- | --- | --- |
| Scaling up (Controlled Vocabulary) | "diffusion of innovation"/ or Organizational Innovation/ | #1 | 40660 |
| Scaling up  (Free text) | ("scal* up" or "scal* out").ab,kf,kw,ti. | #2 | 19681 |
|  | (("scaling" or widespread or spread? or spreading or "rolling out" or "roll out" or "rolls out" or "rolled out" or upscaling or scalability or scalable) adj5 (innovation? or intervention? or technolog* or practice* or care or initiative* or program* or product? or therap* or service* or strateg* OR change? OR proces*)).ab,kf,kw,ti | #3 | 31456 |
|  | ((bring* or brought or taking or take* or increas* or going or implement* or econom*) adj5 scal* adj5 (innovation? or intervention? or technolog* or practice* or care or initiative* or program* or product? or therap* or service* or strateg* OR change? OR proces*)).ab,kf,kw,ti | #4 | 3058 |
| Scaling up  Combined | OR/1-4 | #5 | 91516 |
| Review (Controlled Vocabulary) | meta-analysis/ or systematic review/ or meta-analysis as topic/ or "meta analysis (topic)"/ or "systematic review (topic)"/ or exp technology assessment, biomedical/ | #6 | 221771 |
| Review  (Free text) | ((comprehensive* or environmental or literature* or map* or narrative* or pragmatic* or rapid? or realist? or research? or State-of-the-art or systemati* or umbrella*) adj3 (review* or synthes*)).ti,ab,kf,kw | #7 | 500600 |
|  | ((scoping) adj2 (stud* OR review)).ti,ab,kf,kw | #8 | 5999 |
|  | ((qualitative or quantitative or mixed stud* or mixed method* or meta-narrative*) adj3 (review* or synthes*)).ti,ab,kf,kw | #9 | 12096 |
|  | (research adj3 integrati*).ti,ab,kf,kw | #10 | 2988 |
|  | ((comprehensive* or database* or electronic* or key word* or keyword* or literature* or method* or systemati* or strateg*) adj3 search*).ti,ab,kf,kw | #11 | 194669 |
|  | (Environmental scan* or systematic map* or evidence synthesis* or meta-ethnograph* or overview* or "review of reviews").ti,ab,kf,kw | #12 | 172862 |
|  | (evidence adj2 map*).ti,ab,kf,kw | #13 | 774 |
|  | ((grey* or indexed or published or review* or scan* or scoping* or synthes*) adj3 (literature*)).ti,ab,kf,kw | #14 | 323579 |
|  | (integrative adj3 review*).ti,ab,kf,kw | #15 | 3776 |
|  | (collaborative adj3 review*).ti,ab,kf,kw | #16 | 467 |
|  | (pool* adj3 analy*).ti,ab,kf,kw | #17 | 21817 |
|  | (data synthes* or data extraction* or data abstraction*).ti,ab,kf,kw | #18 | 26409 |
|  | (handsearch* or hand search*).ti,ab,kf,kw | #19 | 9150 |
|  | (mantel haenszel or peto or der simonian or dersimonian or fixed effect* or latin square*).ti,ab,kf,kw | #20 | 25987 |
|  | (meta analy* or metanaly* or meta synthes* or technology assessment* or HTA or HTAs or technology overview* or technology appraisal*).ti,ab,kf,kw | #21 | 179874 |
|  | (meta regression* or metaregression*).ti,ab,kf,kw | #22 | 8687 |
|  | (medline or cochrane or pubmed or medlars or embase or cinahl).ti,ab,hw | #23 | 217048 |
|  | (cochrane or (health adj2 technology assessment) or evidence report).jw | #24 | 19466 |
|  | (comparative adj3 (efficacy or effectiveness)).ti,ab,kf,kw | #25 | 12985 |
|  | (outcomes research or relative effectiveness).ti,ab,kf,kw | #26 | 9220 |
|  | ((indirect treatment or mixed-treatment) adj comparison*).ti,ab,kf,kw | #27 | 704 |
|  | ((examin* or summar* or synthes*) adj3 (evidence* or finding* or literature*)).ti,ab,kf,kw | #28 | 96676 |
| Review  combined | OR/6-28 | #29 | 1007376 |
| Combination of Concepts | 29 AND 5 | #30 | 6820 |
| Limit for comment, conference abstract, etc) | (Comment OR Congress or editorial or Letter).pt | #31 | 1918632 |
| Total Result Without Comment, conference abstracts, etc | 30 NOT 31 | #32 | 6645 |

### Web of Science (2020-08-06)

### Database limit: Social Sciences Citation Index (SSCI) --1900-present ; Science Citation Index Expanded (SCI-EXPANDED) --1900-present; Emerging Sources Citation Index (ESCI) --2005-present search index only

| **Concepts** | **Search strategy keywords** | **Search** | **# Results** |
| --- | --- | --- | --- |
| Scaling up  (Free text) | TS=("scal* up" or "scal* out") | #1 | 46,324 |
|  | TS=(("scaling" or widespread or spread$ or spreading or "rolling out" or "roll out" or "rolls out" or "rolled out" or upscaling or scalability or scalable) NEAR/5 (innovation$ or intervention$ or technolog* or practice* or care or initiative* or program* or product$ or therap* or service* or strateg* OR change$ OR proces*)) | #2 | 71,300 |
|  | TS=((bring* or brought or taking or take* or increas* or going or implement* or econom*) NEAR/5 scal* NEAR/5 (innovation$ or intervention$ or technolog* or practice* or care or initiative* or program* or product$ or therap* or service* or strateg* OR change$ OR proces*)) | #3 | 11,040 |
| Scaling up  Combined | #1 OR #2 OR #3 | #4 | 122,303 |
| Review  (Free text) | TS=((comprehensive* or environmental or literature* or map* or narrative* or pragmatic* or rapid$ or realist$ or research$ or "State-of-the-art" or systemati* or umbrella*) NEAR/3 (review* or synthes*)) | #5 | 643,891 |
|  | TS= ((scoping) NEAR/2 (stud* OR review)) | #6 | 17,310 |
|  | TS=((qualitative or quantitative or "mixed stud*" or "mixed method*" or "meta-narrative*") NEAR/3 (review* or synthes*)) | #7 | 17,993 |
|  | TS=(research NEAR/3 (integrati*)) | #8 | 9,432 |
|  | TS=((comprehensive* or database* or electronic* or "key word*" or keyword* or literature* or method* or systemati* or strateg*) NEAR/3 (search*)) | #9 | 238,121 |
|  | TS=("Environmental scan*" or "systematic map*" or "evidence synthesis*" or "meta-ethnograph*" or overview* or "review of reviews") | #10 | 299,017 |
|  | TS=(evidence NEAR/2 map*) | #11 | 2,052 |
|  | TS=((grey* or indexed or published or review* or scan* or scoping* or synthes*) NEAR/3 (literature*)) | #12 | 391,043 |
|  | TS=(integrative NEAR/3 review*) | #13 | 5,262 |
|  | TS=(collaborative NEAR/3 review*) | #14 | 805 |
|  | TS=(pool* NEAR/3 analy*) | #15 | 35,854 |
|  | TS=("data synthes*" or "data extraction*" or "data abstraction*") | #16 | 25,565 |
|  | TS=(handsearch* or "hand search*") | #17 | 8,050 |
|  | TS=("mantel haenszel" or peto or "der simonian" or dersimonian or "fixed effect*" or "latin square*") | #18 | 40,482 |
|  | TS=("meta analy*" or metanaly* or "meta synthes*" or "technology assessment*" or HTA or HTAs or "technology overview*" or "technology appraisal*") | #19 | 221,013 |
|  | TS=("meta regression*" or metaregression*) | #20 | 10,186 |
|  | TS=(medline or cochrane or pubmed or medlars or embase or cinahl) | #21 | 205,422 |
|  | TS=(comparative NEAR/3 (efficacy or effectiveness)) | #22 | 18,916 |
|  | TS=("outcomes research" or "relative effectiveness") | #23 | 11,596 |
|  | TS=(("indirect treatment" or "mixed-treatment") NEAR/1 comparison*) | #24 | 1,045 |
|  | TS=((examin* or summar* or synthes*) NEAR/3 (evidence* or finding* or literature*)) | #25 | 135,509 |
| Review  combined | #5 OR #6 OR #7 OR #8 OR #9 OR #10 OR #11 OR #12 OR #13 OR #14 OR #15 OR #16 OR #17 OR #18 OR #19 OR #20 OR #21 OR #22 OR #23 OR #24 OR #25 | #26 | 1,394,519 |
| Health and Social Services combined | TS=(care or chiroprati* or clinic* or communit* or dentist* or diagnos* or disease* or drug* or healing* or health* or illness or infection* or injur* or medicin* or medica* or nurs* or nutrition* or optometr* or orthoptic* or patholog* or patient or pharma* or pharmaceutic* or pill* or placebo* or poverty or pregnan* or prevention* or psychiatr* or psycholog* or psychosocial* or remed* or social* or surger* or therap* or treatment* or unemploy* or violence* or wound*) | #27 | 20,482,758 |
| Combination of Concepts | #4 AND #26 AND #27 | #28 | 4,916 |
| Total Result Without “Editorial Material” or “Proceeding paper” OR MEETING ABSTRACT database limit | n/a | #29 | 4,707 |
